# Incidence and outcome of adults with diabetic ketoacidosis admitted to ICUs in Australia and New Zealand

**DOI:** 10.1186/s13054-015-1171-7

**Published:** 2015-12-29

**Authors:** Balasubramanian Venkatesh, David Pilcher, John Prins, Rinaldo Bellomo, Thomas John Morgan, Michael Bailey

**Affiliations:** Intensive Care, Wesley and Princess Alexandra Hospitals, University of Queensland, Brisbane, Queensland Australia; Department of Intensive Care, Alfred Hospital, Melbourne, Victoria Australia; Australian and New Zealand Intensive Care Research Centre, ANZICS Centre for Outcome and Resource Evaluation CORE, Melbourne, Victoria Australia; Endocrinology, University of Queensland, Princess Alexandra Hospital, Brisbane, Queensland Australia; Intensive Care, Australian and New Zealand Intensive Care Research Centre, Melbourne, Victoria Australia; Mater Misericordiae Hospital, Mater Research Institute – UQ, South Brisbane, Brisbane, Queensland Australia; Epidemiologist, Australian and New Zealand Intensive Care Research Centre, Melbourne, Victoria Australia

**Keywords:** Diabetic ketoacidosis, Insulin, Epidemiology, Mortality

## Abstract

**Background:**

Over the last two decades, there have been several improvements in the management of diabetes. Whether this has impacted on the epidemiology and outcome of diabetic ketoacidosis (DKA) requiring intensive care unit (ICU) admission is unknown.

**Method:**

This was a retrospective study of 8533 patients with the diagnosis of DKA admitted to 171 ICUs in Australia and New Zealand between 2000–2013 with separate independent analysis of those on established insulin (Group I) or not on insulin (Group NI) at the time of hospitalisation.

**Results:**

Of the 8553 patients, 2344 (27 %) were identified as NI. The incidence of ICU admission with DKA progressively increased fivefold from 0.97/100,000 (95 % CI 0.84–1.10) in 2000 to 5.3/100,000 (95 % CI 4.98–5.53) in 2013 (*P* < 0.0001), with the proportions between I and NI remaining stable. Rising incidences were observed mainly in rural and metropolitan hospitals (*P* < 0.01). In the first 24 hours in the ICU, mean worst pH increased over the study period from 7.20 ± 0.02 to 7.24 ± 0.01 (*P* < 0.0001), and mean lowest plasma bicarbonate from 12.1 ± 6.6 to 13.8 ± 6.6 mmol/L (*P* < 0.0001). In contrast, mean highest plasma glucose decreased from 26.3 ± 14 to 23.2 ± 13.1 mmol/L (*P* < 0.0001). Hospital mortality was significantly greater in NI as compared to I (2.4 % vs 1.1 %, *P* > 0.0001). Elevated plasma urea in the first 24 hours (≥25 mmol/L, adjusted odds ratio 20.6 (6.54–65.7), *P* < 0.0001) was the strongest individual predictor of mortality.

**Conclusions:**

The incidence of ICU admission of patients with DKA in Australia and New Zealand has increased fivefold over the last decade, with a significant proportion of patients not on insulin at presentation. Overall physiological status in the first 24 hours of ICU admission has progressively improved and mortality rates have remained stable. However, DKA patients not on established insulin therapy at presentation had significantly worse outcomes. This notion has epidemiologic, diagnostic and management implications.

## Background

Type 1 diabetes mellitus affects children and adults worldwide, with an increasing incidence amongst young people [1]. Patients with type 1 diabetes are at risk of developing diabetic ketoacidosis (DKA). The epidemiology of DKA is variable [2–4], and mortality rates range from 2 to 40 % depending on the region [5–12]. In addition, it is now recognized that some patients with type 2 diabetes may also present with DKA. This syndrome, known as ketosis-prone diabetes, was originally described in the African–Americans and Hispanics [13, 14]. It has now been reported in all other races [15–18]. DKA in patients with type 1 diabetes is well characterized. However, the epidemiology, physiological status and outcome of DKA in type 2 diabetes have not been reported in detail; for example, it is likely that these are older patients with co-existing illnesses who as a consequence may have worse outcomes, but the absence of data from large intensive care registries makes this an unknown at any national level.

In the last two decades, diabetes management has undergone paradigm shifts with increased public health education, improved insulin delivery methods, better targeting of oral hypoglycaemic agents and more intensive monitoring [19, 20]. It is likely that these initiatives have resulted in changes in the incidence, early metabolic control or even survival of DKA.

Accordingly, we evaluated trends in epidemiology, metabolic control and mortality in a large cohort of patients admitted to Australian and New Zealand intensive care units (ICUs) with DKA from 2000 to 2013. We hypothesized that metabolic derangements and mortality rates have improved significantly over this period, that patients with DKA not on established insulin therapy at presentation have a worse outcome, and that metabolic abnormalities in the first 24 hours of ICU admission can be identified that help predict mortality.

## Methods

We conducted a retrospective study using data from the Australian and New Zealand Intensive Care Society Adult Patient Database (ANZICS APD) run by the Centre for Outcome and Resource Evaluation. The ANZICS APD is a high-quality bi-national database that captures more than 90 % of all ICU admissions in Australia and New Zealand [21]. The study was approved by the Alfred Hospital Human Research Ethics Committee, Melbourne, Australia, with a waiver of informed consent.

### Description of patients

We included all patients aged over 16 years admitted with a primary diagnosis of DKA to an adult ICU in Australia and New Zealand during a 14-year period from 1 January 2000 to 31 December 2013. ICU readmissions and episodes with no documented mortality outcome were excluded.

We analysed all hospital outcomes (mortality, discharge home, discharge to other hospital, and discharge to rehabilitation). ‘Discharge to rehabilitation’ included discharge to rehabilitation facilities and chronic care facilities such as nursing homes.

The ANZICS APD records the highest and lowest glucose measurements in the first 24 hours of the ICU admission. Patients were classified as those who were on chronic insulin therapy (Group I) prior to admission or not on chronic insulin therapy (Group NI) at presentation. No data on therapy or specific treatments prior to or within the ICU are available in the APD, other than the provision of mechanical ventilation. Standard base excess (SBE) values were calculated from arterial blood gas values (pH and PaCO_2_) recorded for the Acute Physiology and Chronic Health Evaluation (APACHE) II scoring system [22].

Patients were analysed according to the following subgroups: I vs NI diabetes, the degree of SBE derangement, and presence or absence of comorbidities as defined by the APACHE II scores and hospital type. Acute renal failure was defined as present when the 24 hour urine output was <410 ml, or creatinine ≥133 μmol/L and the patient is not receiving chronic dialysis. Hospitals were classified as tertiary, metropolitan, rural/regional or private based on the location of the hospital and the level of care provided. All patients with an admission diagnosis of DKA were analysed to determine annual incidence trends. As previous diabetic status became part of the mandatory minimum dataset in 2007 and was not collected by all hospitals until that time, only those with known prior diabetic status and complete glucose measurements were included in the analysis of factors associated with mortality.

### Statistical analysis

Data are presented as percentages and numbers, means with standard deviations, medians and interquartile ranges (IQRs), or proportions and 95 % confidence intervals (CIs). Chi square tests for equal proportion, *t* tests, or Wilcoxon rank sum tests were used to test for differences as appropriate.

Logistic regression models were used to investigate the change in hospital outcomes over time for all patients with known pre-admission diabetic status, fitting main effects for DKA, year of admission and illness severity, with patients nested within site and site treated as a random effect. To facilitate a measure of patient severity independent of pH, glucose and sodium, and to assess the independent association of these factors to mortality, each patient’s predicted risk of death was calculated in accordance with the Australia and New Zealand risk of death (ANZROD) methodology [23] after separate removal of the pH, glucose, urea, creatinine, potassium and sodium components. ANZROD is an updated mortality prediction model specifically calibrated for use in Australian and New Zealand ICUs. It has been derived from components of the APACHE II and III scoring systems with additional diagnostic variables, and combines eight risk adjustment algorithms, one for each major diagnostic group. It has been shown to have significantly better calibration and discrimination than APACHE III. All data were analysed by SAS Version 9.4 (SAS Institute Inc., Cary, NC, USA). A two-sided *P* value of 0.01 was considered to be statistically significant.

## Results

**Fig. 1:**
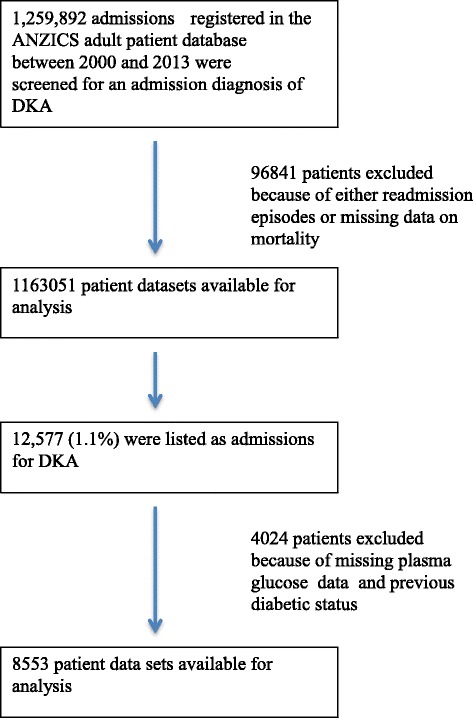
CONSORT flow diagram. *ANZICS* Australian and New Zealand Intensive Care Society, *DKA* Diabetic ketoacidosis

Between January 2000 and December 2013 there were 1,259,892 adult ICU admissions in Australia and New Zealand listed in the ANZICS APD. After exclusion of re-admissions and episodes without documented mortality outcomes, 1,163,051 patient datasets were available for analysis. Of these 12,577 (1.1 %) were listed as admissions for DKA, forming the cohort examined to determine trends in admission to ICU over the study period. Of these, 8553 with documentation of both plasma glucose concentrations and previous diabetic status formed the cohort analysed to determine factors associated with outcome (Fig. 1). Comorbidities were present in 923 patients (11 %).

**Fig. 2:**
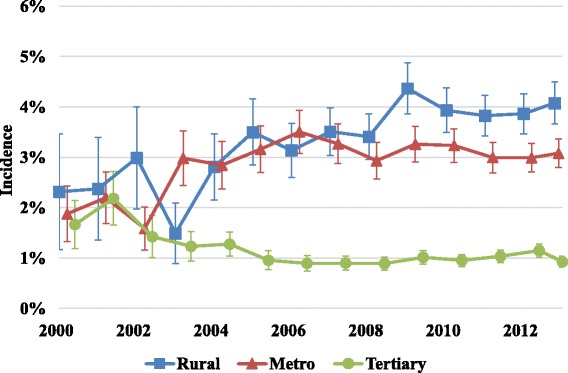
Incidence of ICU DKA admissions across different categories of ICUs. Error bars represent 95 % confidence intervals

The population incidence of ICU admission with DKA progressively increased from 0.97/100,000 (95 % CI 0.84–1.10) in 2000 to 5.3/100,000 (95 % CI 4.98–5.53) in 2013 (*P* < 0.0001). An increasing proportion of DKA admissions relative to total ICU admissions was observed in rural (*P* = 0.0003) and metropolitan hospitals (*P* = 0.01), whilst tertiary centres showed a declining trend (*P* = 0.006) (Fig. 2).

Of the 8553 patients, 2344 (27 %) belonged to the NI group. Overall mean age was 39 ± 18 years with the vast majority originating directly from the Emergency Department. The median duration of time spent in the hospital prior to ICU admission was 4 hours (IQR 1.8–6.5). Overall, 1 % of patients were recorded as having suffered a cardiac arrest and 7 % as requiring mechanical ventilation. There was a reduction in mean APACHE II score from 13.7 ± 6.1 to 12.1 ± 6.8 (*P* < 0.0001), and time spent in the hospital prior to ICU admission decreased from a median of 4.3 hours (IQR 1.54–7.62) to 3.68 hours (IQR 1.83–5.82) (*P* = 0.006).

### Changes in physiological parameters over time

**Fig. 3:**
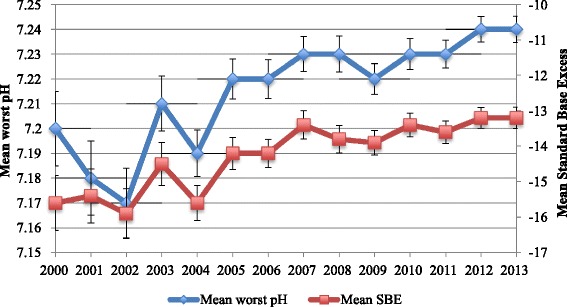
Annual trends in acid–base status. Standard base excess (*SBE*) is expressed in mmol/L. Error bars represent standard errors

There was a clear annual trend towards improved acid–base status and glucose control over the study period. The mean worst pH in the first 24 hours progressively increased from 7.20 ± 0.02 (IQR 7.09–7.3) in 2000 to 7.24 ± 0.01 (IQR 7.15–7.34) in 2013 (*P* < 0.0001) (Fig. 3), the mean lowest plasma bicarbonate increased from 12.1 ± 6.6 to 13.8 ± 6.6 mmol/L (*P* < 0.0001), and the mean SBE increased from –16 ± 8 to –13 ± 8 mmol/L (*P* < 0.0001). The mean highest plasma glucose in the first 24 hours decreased over the study period from 26.3 ± 14.8 to 23.2 ± 13.1 mmol/L (*P* < 0.0001).

### Outcomes

**Fig. 4:**
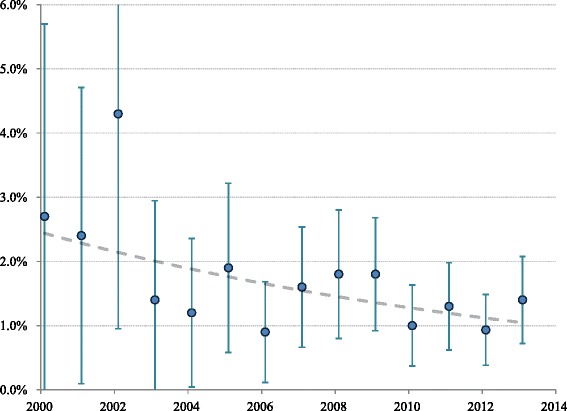
Observed in-hospital mortality (with 95 % confidence interval) of admissions to ICU due to DKA between 2000 and 2013. *P* value for trend for raw mortality = 0.028, unadjusted for declining severity of illness

Median ICU length of stay increased progressively over the study period from 32.7 (IQR 19.8–56.4) to 41.7 (IQR 24.8–67.3) hours (*P* = 0.0004), whereas median hospital length of stay declined from 109 (IQR 72.8–212) to 87 (IQR 50.3–166) hours (*P* < 0.0001). The unadjusted ICU and hospital mortality rates for the entire study period were 0.7 % and 1.4 %, respectively. The increase in mortality between ICU and hospital discharge was confined almost exclusively to the NI group of patients. Although we observed a reduction in raw mortality (Fig. 4), there was no change in risk-adjusted mortality during the 14-year study period after accounting for declining severity of illness. Analysis of survival trends over three different epochs (2000–2004, 2005–2009, 2010–2013) did not identify any differences in mortality. About 98 % of the I cohort and patients and 96 % of the NI cohort were discharged home, the remainder to a rehabilitation or chronic care facility.

### Comparison of Group I and Group NI DKA

Patients in Group I were younger with a lower prevalence of chronic co-morbidities such as liver disease, immune-suppression and malignancies. This group had better glucose control and better renal function in the first 24 hours, while those in the NI group had less severe disorders of acid–base status. Mortality and ICU and hospital lengths of stay were significantly higher in the NI cohort patients (Table 1).

**Table 1:** Comparison of diabetic ketoacidosis in patients on established chronic insulin therapy (I) vs. not on chronic insulin therapy (NI)

	I diabetes (n = 6209)	NI diabetes (n = 2344)	*P* value
Age (years)	36.68 (17.35)	44.89 (19.61)	<0.0001
APACHE II score	39 (27–54)	45 (31–61)	<0.0001
Weight (kg)	69.34 (18.26)	76.75 (23.66)	<0.0001
Chronic respiratory disease	1 % (90)	2 % (41)	0.31
Chronic cardiovascular disease	3 % (188)	4 % (87)	0.11
Chronic liver disease	0 % (30)	2 % (36)	<0.0001
Chronic renal failure	3 % (169)	3 % (60)	0.68
Immune disease	0 % (29)	1 % (27)	0.0005
Immunosuppressed by therapy	1 % (54)	2 % (49)	<0.0001
Metastases	0.3 % (20)	1.4 % (32)	<0.0001
Leukaemia	0.1 % (7)	0.5 % (11)	0.001
Cardiac arrest in the previous 24 hours	1 % (37)	1 % (14)	0.99
Physiological derangement			
Mean arterial pressure (mmHg)	87.56 (22.93)	89.74 (24.37)	0.0001
Mechanical ventilation in first 24 hours	6 % (385)	9 % (204)	<0.0001
Glasgow Coma Score	14.16 (2.17)	13.96 (2.43)	0.0002
Acute renal failure	5 % (292)	7 % (162)	<0.0001
24 hour urine output (mL)	2468 (1500–3700}	2505 (1590–3800)	0.003
Laboratory derangements			
Arterial pH	7.24 (7.12–7.32)	7.27 (7.16–7.36)	<0.0001
Arterial PCO_2_ (mmHg)	29 (23–35)	31 (24–38)	<0.0001
Bicarbonate (mmol/L)	13.82 (6.73)	15.56 (7.57)	<0.0001
Standard base excess (mEq/L)	−14.51 (8.43)	−12.07 (9.27)	<0.0001
PaO_2_ (mmHg)	104.2 (59.88)	106.38 (63.9)	0.2
Sodium (mmol/L)	137.88 (7.25)	139.22 (9.68)	<0.0001
Potassium (mmol/L)	4.4 (3.6–5)	4.2 (3.4–4.8)	<0.0001
Urea (mmol/L)	6.8 (4.3–11.6)	7.6 (4.5–14.8)	<0.0001
Creatinine (μmol/l)	91 (55–136)	100 (65–152)	<0.0001
Albumin (mmol/L)	31.16 (6.8)	30.43 (6.73)	<0.0001
Bilirubin (mmol/L)	8 (5–14)	8 (5–13)	0.96
Highest glucose (mmol/L)	23.5 (13.07)	24.29 (14.35)	0.015
Lowest glucose (mmol/L)	7.1 (5.2–10)	8 (5.6–11.7)	<0.0001
Outcomes			
ICU mortality^a^	0.6 % (34)	1.1 % (26)	0.006
Hospital mortality	1.1 % (67)	2.4 % (56)	<0.0001
ICU length of stay (hours)	41.6 (24–64.2)	46.5 (26.8–73.7)	<0.0001
Hospital length of stay (days)	3.75 (2.39–6.74)	5.12 (3.03–9.65)	<0.0001

Values represent worst recorded in first 24 hours of ICU admission unless stated

^a^Deaths during the initial admission to ICU

*APACHE* Acute Physiology and Chronic Health Evaluation, *ICU* Intensive care unit

Figures in brackets indicate actual numbers or range or SD as appropriate

### Comparison of survivors and non-survivors

Non-survivors were older and more likely to have experienced cardiac arrest and mechanical ventilation (Table 2). They had lower Glasgow Coma Score values, higher plasma glucose concentrations, higher arterial PCO_2_ and lower pH values, and higher plasma urea and creatinine concentrations. However, there was no difference in the degree of overall metabolic acidosis as evidenced by SBE values (Tables 3, 4, 5, 6 and 7). Those who died also had a longer duration of stay in ICU and hospital. Using multivariable logistic regression analysis, the strongest individual independent predictor of mortality was a high plasma urea concentration. For those with elevated plasma urea >25 mmol/L, the adjusted odds ratio for death was 20.6 (95 % CI 6.4–65.7) (*P* < 0.0001) (Table 7).

**Table 2:** Comparison of survivors and non-survivors with diabetic ketoacidosis

Label	Alive	Died	*P* value
	n = 8430	n = 123	
“I” diabetes	73 % (6142)	54 % (67)	<0.0001
Age (years)	38.56 (18.13)	64.14 (16.93)	<0.0001
APACHE II score	44.31 (23.17)	94.5 (34.95)	<0.0001
Weight (kg)	71.23 (20.04)	71.33 (20)	0.99
Chronic respiratory disease	1 % (118)	11 % (13)	<0.0001
Chronic cardiovascular disease	3 % (257)	15 % (18)	<0.0001
Chronic liver disease	1 % (62)	3 % (4)	0.002
Chronic renal failure	3 % (217)	10 % (12)	<0.0001
Immune disease	1 % (53)	2 % (3)	0.013
Immunosuppressed by therapy	1 % (97)	5 % (6)	0.0002
Metastases	1 % (44)	7 % (8)	<0.0001
Leukaemia	0.2 % (15)	2.4 % (3)	<0.0001
Cardiac arrest in the previous 24 hours	1 % (41)	9 % (10)	<0.0001
ICU admission source: operating theatre	1 % (48)	1 % (1)	0.72
ICU admission source: emergency	87 % (7361)	82 % (101)	0.09
ICU admission source: ward	4 % (307)	11 % (14)	<0.0001
Physiological derangement			
Mean arterial pressure (mm Hg)	88.32 (23.22)	77.13 (28.88)	<0.0001
Mechanical ventilation in first 24 hours	6 % (537)	42 % (52)	<0.0001
Glasgow Coma Score	14.16 (2.14)	10.13 (4.56)	<0.0001
Acute renal failure	5 % (419)	28 % (35)	<0.0001
24 hour urine output (mL)	2540 (1600–3800)	1335 (515–1953)	<0.0001
Laboratory derangements			
Arterial pH	7.22 (0.16)	7.18 (0.2)	0.004
Arterial PCO_2_ (mmHg)	29.67 (11.16)	34.36 (11.19)	<0.0001
Bicarbonate (mmol/l)	14.28 (7.01)	16.07 (7.4)	0.006
Standard base excess (mEq/L)	−13.83 (8.74)	–13.2 (8.69)	0.46
PaO_2_ (mmHg)	114.46 (67)	134.47 (96.1)	0.003
Sodium (mmol/L)	138 (135–142)	142 (136–147)	<0.0001
Potassium (mmol/L)	4.37 (1.07)	4.75 (1.23)	0.0001
Urea (mmol/L)	6.9 (4.3–12.1)	17.3 (11.9–25.55)	<0.0001
Creatinine (μmol/L)	93 (57–140)	191 (120–267)	<0.0001
Albumin (mmol/L)	31.0 (6.76)	26.1 (7.08)	<0.0001
Bilirubin (mmol/L)	8 (5–14)	11 (6–19)	0.02
Highest glucose (mmol/L)	23.6 (13.31)	32.3 (18.73)	<0.0001
Lowest glucose (mmol/L)	7.4 (5.3–10.3)	9.6 (6.1–15.3)	<0.0001
Haematocrit	0.38 (0.08)	0.34 (0.09)	<0.0001
White cell count	14.6 (10.2–20.6)	16.8 (11.5–22.5)	0.04
Length of stay outcomes			
ICU length of stay (hours)	42.8 (24.7–66.8)	66.1 (31.3–156.2)	<0.0001
Hospital length of stay (days)	4.01 (2.58–7.41)	5.75 (2.04–14.86)	0.022

Values represent worst recorded in first 24 hours of ICU admission unless stated

*APACHE* Acute Physiology and Chronic Health Evaluation, *I* Insulin, *ICU* Intensive care unit

Figures in brackets indicate actual numbers or range or SD as appropriate

**Table 3:** Multivariable logistic regression model for factors associated with in-hospital mortality: highest glucose in first 24 hours

	Adjusted odds ratio	(95 % CI)	*P* value
ANZROD	1.12	(1.10–1.15)	<0.001
Highest glucose in first 24 hours (mmol)			0.002
<10	1.00	Reference category	
10–19.9	0.33	(0.16–0.7)	
20–29.9	0.49	(0.23–1.03)	
30–39.9	1.06	(0.50–2.26)	
≥40	0.98	(0.47–2.06)	

*ANZROD* Australian and New Zealand risk of death model with glucose components removed, *CI* Confidence interval

**Table 4:** Multivariable logistic regression model for factors associated with in-hospital mortality: lowest pH in first 24 hours

	Adjusted odds ratio	(95 % CI)	*P* value
ANZROD	1.13	(1.11–1.15)	<0.001
Lowest pH in first 24 hours (mmol)			0.31
≥7.35	1.00	Reference category	
7.30–7.349	0.79	(0.38–1.65)	
7.20–7.29	0.53	(0.27–1.06)	
7.10–7.19	1.14	(0.60–2.15)	
<7.10	0.79	(0.43–1.47)	

*ANZROD* Australian and New Zealand risk of death model with pH components removed, *CI* Confidence interval

**Table 5:** Multivariable logistic regression model for factors associated with in-hospital mortality: highest sodium in first 24 hours)

	Adjusted odds ratio	(95 % CI)	*P* value
ANZROD	1.12	(1.10–1.14)	<0.001
Highest sodium in first 24 hours (mmol)			0.001
<130	3.32	(1.35–8.19)	
130–134	1.50	(0.82–2.72)	
135–144	1.00	Reference category	
145–154	2.57	(1.56–4.23)	
≥155	2.17	(1.01–4.66)	

*ANZROD* Australian and New Zealand risk of death model with sodium component removed, *CI* Confidence interval

**Table 6:** Multivariable logistic regression model for factors associated with in-hospital mortality: highest potassium in first 24 hours)

	Adjusted odds ratio	(95 % CI)	*P* value
ANZROD	1.13	(1.11–1.15)	<0.001
Highest potassium in first 24 hours (mmol)			0.040
<3.0	0.00	(0.00–1.06)	
3.0–3.4	0.83	(0.20–3.37)	
3.5–4.9	1.00	Reference category	
5.0–5.9	1.99	(1.27–3.10)	
≥6.0	1.59	(0.83–3.04)	

*ANZROD* Australian and New Zealand risk of death model with potassium component removed, *CI* Confidence interval

**Table 7:** Multivariable logistic regression model for factors associated with in-hospital mortality: urea and creatinine)

	Adjusted odds ratio	(95 % CI)	*P* value
ANZROD	1.10	(1.08–1.12)	<0.0001
Urea (mmol)			<0.001
<6	1.00	Reference category	
6–9.9	5.58	(2.01–15.5)	
10–14.9	10.4	(3.56–30.7)	
15–24.9	11.4	(3.79–34.6)	
≥25	20.6	(6.54–64.7)	
Creatinine (μmol)			0.36
<100	1.00	Reference category	
100–129	0.62	(0.28–1.37)	
130–159	0.95	(0.43–2.12)	
160–219	1.45	(0.70–2.99)	
≥220	1.22	(0.57–2.62)	

*ANZROD* Australian and New Zealand risk of death model with urea and creatinine components removed, *CI* Confidence interval

### Sensitivity analyses

Trends in metabolic status (pH, SBE, glucose), illness severity, length of stay and mortality were analysed after stratifying for hospital category and size. In addition, we analysed all outcomes in the subgroup of ICUs that continuously contributed to the ANZICS APD. Findings were similar to those noted in the overall cohort of patients, with no evidence of differences in outcome across different hospital categories or sizes.

Given the large size of the study cohort, we also examined the database for missing data and, in such instances, additional sensitivity analyses were performed whereby all patients with missing data were included in the analysis as a separate variable. The proportions missing were very small for serum sodium (2.6 %), potassium (2.7 %), urea (1 %) and creatinine (3 %). The results from the additional sensitivity analyses were concordant with those from the original results. The proportion missing for pH was 23.4 %. Nevertheless, there was no significant relationship between pH and mortality in this cohort regardless of whether missing patients were included or not.

A comparison of the two population cohorts (those included in and those excluded from the analysis) revealed no statistically significant differences in demographics and APACHE II scores, proportion of patients on established insulin therapy, highest glucose, lowest plasma bicarbonate, and urea (*P* = not significant) and clinically insignificant differences in pH (7.25 vs 7.23) and PCO_2_ (30 vs 29) (*P* < 0.0001), suggesting that the cohort included in the analysis is representative of the entire population.

## Discussion

### Key findings

We studied the epidemiology, early physiological features, and outcomes of patients admitted to Australia and New Zealand ICUs with the diagnosis of DKA over more than a decade. We found a fivefold increase in the incidence of ICU admission and demonstrated that more than a quarter of patients were not on insulin at presentation. As a proportion of overall ICU admissions, DKA increased progressively in rural and metropolitan ICUs but decreased in tertiary centres, while physiological status in the first 24 hours of ICU admission improved significantly. Moreover, although overall ICU and hospital mortality remained low, patients not on insulin at presentation (the NI cohort) had a worse outcome compared to those with a history of prior insulin therapy (the I cohort). Finally, an elevated plasma urea concentration was strongly independently associated with mortality.

### Comparison with previously studies

We identified a change in epidemiology with an increased population incidence of ICU admission of DKA. This may reflect multiple factors. These include the rising population incidence of diabetes in Australia and New Zealand [24, 25], the widespread availability via government subsidy of glucometers which also measure ketones and facilitate earlier hospital presentation, the introduction of requirements for mandatory rapid patient processing in Australian Emergency Rooms [26], and more liberal and frequent ICU admission for further resuscitation and stabilization. Finally, the increased use of short-acting prandial insulin analogues and basal-bolus regimes may have also contributed by reducing the “overlap” in insulin actions, thus increasing the possibility of insulinopaenia, especially if a dose is missed (a common occurrence) [27].

More than a quarter of our ICU patients with DKA had no history of prior insulin therapy. The absence of pre-existing insulin therapy suggests two possible diagnoses: a) type 2 diabetes-associated DKA or b) a first presentation of type 1 diabetes-associated DKA. However, the older age in the NI cohort combined with the greater prevalence of comorbidities and the higher mortality despite less severe acid–base derangements is strongly suggestive of the former. This is also consistent with the changing epidemiology of type 2 diabetes, the incidence of which is rising [24], with patients diagnosed at a younger age and living longer due to improved cardiovascular outcomes. The longer disease duration means many more patients are treated with insulin [24, 27] and have reduced endogenous insulin secretory capacity. The resultant endogenous insulinopaenia may make them more “like” type 1 diabetes, and hence prone to DKA.

An increasing proportion of patients with DKA were admitted to rural and regional ICUs. This phenomenon may in part reflect improved ICU staffing with certified intensive care specialists in peripheral units [28]. This may also reflect a lack of high dependency unit facilities in smaller centres, fewer available general and endocrine physicians and less favourable nurse–patient ratios on the medical floor. In addition, over the study period the proportion of rural ICUs increased by 312 %, metropolitan ICUs by 230 % and tertiary ICUs by 135 %. These structural factors could account for the trend towards greater increase in the incidence in rural and metropolitan centres and may also account for the progressive increase in ICU length of stay noted in our study, similar to reports in epidemiological studies of DKA in the USA [29].

We found a low mortality rate. DKA mortality rates in other studies have ranged from 2–40 %, although comparison is limited by their small sample sizes (all <250 patients) [7–12, 30–33] and the fact that not all patients were admitted to ICU. Most were single centre studies dating back to the early 1990s and may not reflect more recent developments in diabetes. Our mortality data also compare favourably with those from two other large national non-ICU registries from Denmark [6] and the USA (n = 15,994) [29]. A large Italian registry comprising >250,000 acute hyperglycaemic complications reported annual trends and a step-wise reduction in hospitalisation for diabetic complications, but did not provide specific data for DKA [3]. An Australian tertiary centre study [34] of trends in deaths associated with hyperglycaemic emergencies between 1973 and 1988 reported a mortality rate of 4.9 % in patients with DKA but also did not focus on ICU patients.

Our study provides evidence that DKA-related ICU and hospital mortality has remained steady for nearly a decade and a half in conjunction with improved metabolic control. The secular trend in improved acid–base status and glucose values on ICU day 1 could be attributed to several factors including earlier presentation, improved resuscitation in Emergency Departments and subsequently in ICU, plus a background of better diabetic management in the community. The question remains as to why, despite an improved physiological status over time, no improvement in mortality was observed. The hospital mortality in DKA is very low to start with, about 1.5 %. To demonstrate an improvement in mortality with such low baseline mortality rates is very difficult. Moreover, this is a retrospective study and therefore difficult to adjust for a number of confounding variables. Although there is a rising incidence of type 2 diabetes in the community and therefore a tendency for worse outcomes [24, 25], our database did not capture information on the type of diabetes and therefore it is difficult to attribute the lack of a decline in mortality to an increasing proportion of type 2 diabetes. Moreover, neither the mean age nor the body weight or chronic cardiovascular disease showed an increasing trend over the study period to suggest a greater proportion of type 2 diabetes.

Elevated urea concentrations stood out above glucose, sodium and potassium concentrations as a strong independent predictor of mortality. Although plasma urea is dependent on both renal function and pre-renal factors such as hydration and catabolic status, its strong relationship with mortality in this dataset appeared independent of renal function. This is in conjunction with the finding of sodium >155 mmol/L associated with an odds ratio for death >2 (Table 5), suggesting that dehydration may be an important marker of illness severity. The other possible explanation is that elevated urea is also a sign of protein catabolic state which may be seen in severe DKA. Also urea elevation out of proportion to creatinine could indicate gastrointestinal bleeding secondary to stress ulceration suggesting a severe form of DKA.

### Implications of study findings

Our ICU data demonstrate significant changes in the epidemiology, management and some outcomes of DKA in Australia and New Zealand, with several factors potentially driving such changes. As discussed, the sizeable proportion of ICU DKA patients not on insulin at presentation were at greater risk of unfavourable outcomes despite less severe acid–base disturbances, suggesting that the majority had type 2 diabetes. On the other hand, some patients in the group who were receiving insulin prior to ICU admission may also have had type 2 diabetes. Although speculative in both cases, the clearest message is that ICU patients admitted with DKA who were not receiving insulin prior to admission need particular attention in view of their greater risk. It is noteworthy in this context that newer drugs for diabetes management such as the sodium–glucose co-transporter-2 (SGLT2) inhibitors, which promote glucose loss by the kidney, may predispose patients with type 2 diabetes to DKA [35]. Our findings from Australia and New Zealand ICUs should be generalisable to other developed healthcare systems including Europe and the USA. Finally, the observation that elevated urea concentrations have prognostic implications suggests a possible application in patient triage.

### Strengths and weaknesses of the study

A unique strength of the present study is the size of the analysed datasets, originating from a large bi-national registry of DKA spanning nearly a decade and a half. To our knowledge, this is the first report of its kind to present not only epidemiological data but changes over more than a decade in metabolic indices, management and outcomes. Furthermore, the database is highly representative, by 2012 encompassing over 90 % of all ICU admissions in Australia and New Zealand. With all data collected prospectively for routine quality surveillance purposes, the size of the cohort ensures a robust annual analysis of biochemical status and mortality rates as recently shown in the assessment of sepsis outcomes [36].

Our study has some limitations. The ANZICS APD does not record serum or urine ketone concentrations and does not specify diagnostic criteria for DKA, relying on treating clinicians to apply accepted standard definitions. In clinical practice, however, the diagnosis of DKA is often made in the context of the appropriate clinical setting and the presence of a raised serum or urine ketone concentration, such that the diagnosis is rarely a difficult diagnostic challenge. Although data are mostly recorded by trained collectors, the specific accuracy of diagnostic coding for DKA has not been independently verified. The APD only records the primary diagnosis and chronic health conditions, thus acute co-morbidities are not known. This is important as many DKAs might not be coded because DKA might not be considered the primary diagnosis. For example a post-operative patient with type 2 diabetes and severe abdominal sepsis (due to a perforation) may present with both conditions, but ICU staff would only code the surgical condition. Given the unknown confounding influence of acute co-morbid conditions, there could be an underestimation of the true incidence of DKA. However, the ANZICS APD is recognized as a high-quality clinical registry [21] with extensive data validation rules and on-site audits of data quality.

The database in its current format does not capture information on therapy other than the provision of mechanical ventilation. Although the presence of established insulin therapy is collected at the time of admission, categorization of patients as type 1 or type 2 diabetes mellitus is not available. Also data on fluid, insulin and other therapies were unavailable. Moreover, serum lactate concentrations were not available for analysis as they are not captured as part of the APACHE II score. Therefore, it is difficult to decipher precisely the burden of metabolic acidosis from ketones and non-ketone anions. Finally, there was no information on the metabolic status and outcomes of patients admitted directly to the medical wards with DKA. Despite our data demonstrating a rising incidence of ICU DKA admissions, the overall incidence of DKA admissions to hospital is unknown given this absence of data.

## Conclusions

The incidence of admission to Australia and New Zealand ICUs with DKA has increased fivefold over more than a decade, with over a quarter of patients not on established chronic insulin therapy at presentation and carrying a significantly worse outcome. A sizeable proportion of patients not on prior insulin could represent DKA associated with type 2 diabetes. Several markers of physiological status in the first 24 hours of ICU admission have improved progressively, with overall mortality rates remaining stable and low. The presence of a raised plasma urea concentration was strongly predictive of mortality. These findings provide insights into the potential importance of prior insulin history and presenting plasma urea concentrations for risk stratification.

## Key messages

- The present study examined changes in the epidemiology, national burden, management and outcomes of severe diabetic ketoacidosis (DKA) requiring ICU admission across 171 ICUs in Australia and New Zealand, spanning over a decade and a half (2000–2013).
- Our data demonstrate significant changes in the epidemiology, management and some outcomes of DKA in Australia and New Zealand, with several factors potentially driving such changes.
- The demonstration that patients who are not on established insulin therapy represent a quarter of such patients and are at greater risk of unfavourable outcomes implies that such patients do develop DKA and require specific attention. This notion has important epidemiologic, diagnostic and management implications

## Abbreviations

ANZICS APD: Australian and New Zealand Intensive Care Society Adult Patient Database
ANZROD: Australia and New Zealand risk of death
APACHE: Acute Physiology and Chronic Health Evaluation
CI: Confidence interval
DKA: Diabetic ketoacidosis
I: Patients on insulin
ICU: Intensive care unit
IQR: Interquartile range
NI: Patients not on insulin
SBE: Standard base excess
